# Effects of oxonic acid-induced hyperuricemia on mesenteric artery tone and cardiac load in experimental renal insufficiency

**DOI:** 10.1186/s12882-015-0033-5

**Published:** 2015-03-27

**Authors:** Venla Kurra, Tuija Vehmas, Arttu Eräranta, Jarkko Jokihaara, Päivi Pirttiniemi, Heikki Ruskoaho, Heikki Tokola, Onni Niemelä, Jukka Mustonen, Ilkka Pörsti

**Affiliations:** Department of Internal Medicine, School of Medicine, University of Tampere, FIN-33014 Tampere, Finland; Department of Hand Surgery, Tampere University Hospital, Tampere, Finland; Department of Pharmacology and Toxicology, Institute of Biomedicine, University of Oulu, Oulu, Finland; Division of Pharmacology and Pharmacotherapy, Faculty of Pharmacy, University of Helsinki, Helsinki, Finland; Department of Pathology, Oulu University Hospital, Oulu, Finland; Department of Clinical Chemistry, Seinäjoki Central Hospital Laboratory, Seinäjoki, Finland; Department of Internal Medicine, Tampere University Hospital, Tampere, Finland

**Keywords:** Uric acid, Artery tone, Experimental chronic renal insufficiency, 5/6 nephrectomy, Oxonic acid

## Abstract

**Background:**

Recent studies suggest a causal role for increased plasma uric acid in the progression of chronic renal insufficiency (CRI). However, uric acid also functions as an antioxidant with possible beneficial effects.

**Methods:**

We investigated the influence of hyperuricemia on mesenteric arterial tone (main and second order branch) and morphology in experimental CRI. Forty-four Sprague–Dawley rats were 5/6 nephrectomized (NX) or Sham-operated and fed 2.0% oxonic acid or control diet for 9 weeks.

**Results:**

Oxonic acid feeding elevated plasma uric acid levels 2.4 and 3.6-fold in the NX and Sham groups, respectively. Plasma creatinine and urea were elevated 2-fold and blood pressure increased by 10 mmHg in NX rats, while hyperuricemia did not significantly influence these variables. Right and left ventricular weight, and atrial and B-type natriuretic peptide mRNA content were increased in NX rats, but were not affected by hyperuricemia. In the mesenteric artery, hyperuricemia did not influence vasoconstrictor responses *in vitro* to norepinephrine or potassium chloride. The small arteries of NX rats featured hypertrophic remodeling independent of uric acid levels: wall to lumen ratio, wall thickness and cross-sectional area were increased without changes in lumen diameter. In the main branch, vasorelaxations to acetylcholine were impaired in NX rats, but were not affected by hyperuricemia. In contrast, relaxations to the large-conductance Ca^2+^-activated K^+^-channel (BK_Ca_) opener NS-1619 were reduced by oxonic acid feeding, whereas responses to nitroprusside were not affected.

**Conclusions:**

Experimental hyperuricemia did not influence cardiac load or vascular remodeling, but impaired BK_Ca_ -mediated vasorelaxation in experimental CRI.

## Background

The prevalence of increased plasma uric acid (UA), hyperuricemia, is high in patients with chronic renal insufficiency (CRI). The detrimental effects of hyperuricemia have been linked to cardiovascular complications, as high plasma UA levels commonly predict the development of hypertension [1] and the loss of renal function [2]. To date, however, the contribution of UA to cardiovascular disease has still remained controversial [3].

Previous experimental studies, carried out in rats made hyperuricemic by the inhibition of the UA degrading enzyme uricase using dietary 2.0% oxonic acid, have suggested a causal relationship between high UA and cardiovascular disease [4-7]. UA has been associated with stimulation of the renal renin-angiotensin system (RAS) and reduced nitric oxide (NO) synthesis. These mechanisms may have participated in the subsequent hypertrophic remodeling of the preglomerular arteries, tubulointerstitial damage, and thus predisposed to enhanced sodium retention [4-6,8]. Previously, incubation of the rat aortic rings with UA was found to reduce vasodilatation in response to acetylcholine (Ach) [9], while the endothelial NO production *in vitro* was reduced in hyperuricemic rats [10]. This suggests that the detrimental effects of hyperuricemia may partly result from endothelial dysfunction.

There is an ongoing debate about the role of UA in vascular disease. This arises from the ability of UA to reduce oxidative stress by preventing the superoxide radical from reacting with NO to generate peroxynitrite [11]. Peroxynitrite can impair NO-mediated relaxation by inhibiting a critical cofactor of the endothelial NO synthase [12], while it also can modulate vascular tone via smooth muscle [13]. In the aortas of apolipoprotein E-deficient mice, the ability of UA to reduce peroxynitrite levels was associated with improved Ach-elicited relaxation [12]. This result is in agreement with our earlier report that oxonic acid-induced hyperuricemia improved NO-mediated relaxation of the carotid artery in experimental CRI by alleviating oxidative stress [14].

In experimental CRI, reduced vasodilatation via Ca^2+^-activated K^+^-channels (BK_Ca_), observed in isolated mesenteric arterial branches, may precede the elevation of blood pressure (BP) [15,16]. However, until now the vascular effects of UA have only been studied in arteries in which the endothelium-mediated dilatation is mainly mediated via NO [12,14]. Here we examined the tone of mesenteric arteries *in vitro* from the 5/6 nephrectomized (NX) and Sham-operated rats allocated to 2.0% oxonic acid diet for 9 weeks. Our findings suggest that oxonic acid diet impaired relaxation via BK_Ca_ in arterial smooth muscle, but did not significantly affect the endothelium-dependent responses, resistance artery structure, or cardiac load in experimental CRI.

## Methods

### Animals and experimental design

Male Sprague–Dawley rats (weight 335–341 g) were housed in standard animal laboratory conditions with free access to water and food pellets (Lactamin R34, AnalyCen, Lindköping, Sweden) containing 0.9% calcium, 0.8% phosphorus, 0.27% sodium, 0.2% magnesium, 0.6% potassium, 1500 IU/kg vitamin D and 12550 kJ/kg energy, 16.5% protein, 4.0% fat, 58% nitrogen-free extract, 3.5% fiber, 6.0% ash, and 10% water. At the age of 8 weeks (study week 0), the rats were anesthetized with ketamine/diazepam (75 and 2.5 mg/kg, intraperitoneally, respectively) and NX (n = 22) was carried out by the removal of upper and lower poles of the left kidney and the whole right kidney. The sham-operation (n = 22) was performed by kidney decapsulation as previously described [15,17]. Antibiotics (metronidazole 60 mg/kg and cefuroxim 225 mg/kg) and pain killers (buprenorphine 0.2 mg/kg subcutaneously) were given post-operatively three times a day for the total duration of three days. Three weeks after the surgery (study week 3, i.e. preceding the treatments), the rats were divided into groups (Sham, Sham+Oxo, NX, NX+Oxo) so that systolic BPs, body weights and urine volumes were similar in the two Sham and two NX groups. Oxonic acid (Oxo; 20 g/kg chow, Sigma-Aldrich Chemical Co, St Louis, MO, USA) was then added in the chow of the Sham+Oxo and NX+Oxo groups, whereas the Sham and NX groups continued on the normal chow. Hyperuricemia was confirmed by tail vein sampling at study week 5, and 24-hour urine was collected in metabolic cages at the end of the 2nd and 11th study weeks [14]. Before and during the treatment period systolic BP was measured at 28°C by the tail-cuff method as the averages of five recordings in each rat (Model 129 Blood Pressure Meter; IITC Inc., Woodland Hills, CA, USA). The animals were kept in plastic restrainers during the measurements. To increase the reliability, before the actual measurements the rats were preconditioned on two separate occasions.

After 9 weeks of diets, the rats were weighed and anesthetized (urethane 1.3 g/kg) and blood samples from carotid artery for plasma creatinine, urea and UA were drawn in to chilled tubes with EDTA and heparin as anticoagulants. Blood samples from 2 rats in the Sham group were lost due to technical problems. Plasma creatinine and urea were measured using standard clinical chemical methods (Cobas Integra 800 Clinical Chemical Analyzer, Roche Diagnostics, Basel, Switzerland). UA was measured using an enzymatic colorimetric method [18]. The hearts and the kidneys were removed and weighed. The ventricles were snap frozen in liquid nitrogen-cooled isopentane and stored at −70°C until the extraction of the total RNA. The experimental design of the study was approved by the Animal Experimentation Committee of the University of Tampere, Finland, and the Provincial Government of Western Finland Department of Social Affairs and Health, Finland. The investigation conforms to the Guide for the Care and Use of Laboratory Animals published by the US National Institutes of Health (NIH Publication No. 85–23, revised 1996).

### Functional responses and morphology of the mesenteric arterial preparations in vitro

The experiments on isolated arteries were performed from 10 randomly chosen rats in each group. Two successive (3 mm in length) sections from the main branch of superior mesenteric artery were excised beginning 3 mm distally from the mesenteric artery-aorta junction, and small (1.9 mm in length) second order branches were dissected from the mesenteric arterial bed under a microscope (Nikon SMZ-2 T, Nikon Inc., Japan). The endothelium was removed mechanically from the proximal piece of the large artery and from one small arterial ring by perfusing air through the lumen [15,17]. In the distal piece of the main branch and in the other small ring the endothelium was left intact. The large arterial rings were equilibrated in a resting preload of 4.905 mN/mm [16], while the small arterial preparations were normalized so that the internal diameter of the vessel was set at 90% of that obtained when exposed to intraluminal pressure of 100 mmHg in the relaxed state [15,17]. In the large arteries, the force of contraction was measured with isometric force-displacement transducer and registered on a polygraph (FT 03 transducer, 7E Polygraph; Grass Instrument Co., Quincy, MA, USA) and in the small arteries the computerized Mulvany multimyograph (Model 610A, J.P. Trading, Aarhus, Denmark) was employed [16]. All arterial preparations were kept in physiological salt solution (PSS, pH 7.4) containing (mM): NaCl 119.0, NaHCO_3_ 25.0, glucose 11.1, CaCl_2_ 1.6, KCl 4.7, KH_2_PO_4_ 1.2, MgSO_4_ 1.2, and aerated with 95% O_2_ and 5% CO_2_ at 37°C.

Contractions were cumulatively elicited in response to norepinephrine (NE) and KCl in large and small arterial rings. The main branch of the mesenteric artery was chosen for the relaxation responses due to our extensive previous experience with this model [19-22]. Vasodilatation to Ach was investigated in endothelium-intact rings after precontractions with 5 μM NE in the absence and presence of the non-specific nitric oxide synthase (NOS) inhibitor N^G^-nitro-L-arginine methyl ester (L-NAME, 0.1 mM). Responses to the NO donor sodium nitroprusside (NP) and BK_Ca_ opener NS-1619 were studied in endothelium-denuded rings precontracted with KCl [23]. The efficiency of the removal of the endothelium was confirmed by the lack of relaxation to Ach [22]. During the experimental protocol, the rings were allowed a 30 min period at baseline tension in between each of the concentration-response challenges.

Morphology of small arteries at 90 mmHg intraluminal pressure was examined using a pressure myograph (Living Systems Instrumentation, Inc., Burlington, VT, USA), as previously reported [24]. The development of myogenic tone was inhibited by Ca^2+^-free solution containing 30 mmol/L EDTA [25]. Small arteries were chosen for the study of morphology due to their contribution to peripheral vascular resistance via remodeling [26].

### Ventricular atrial and B-type natriuretic peptide, skeletal α-actin and ß–myosin heavy chain mRNAs (ANP, BNP, SkαA and β-MHC, respectively)

Total RNA was isolated from the ventricles by the guanidine thiocyanate CsCl method, and 20-μg samples of RNA were transferred to nylon membranes (Osmonics) for Northern blot analysis as described previously [27,28]. Full-length rat ANP cDNA probe (a gift from Dr. Peter Davies, Queen’s University, Kingston, Canada), and cDNA probes for rat BNP, SkαA, ß-MHC and 18S were prepared as previously reported [27,28]. The cDNA probes were labeled, the membranes were hybridized and washed, and exposed with PhosphorImager screens (Amersham Biosciences), which were scanned with Molecular Imager FX Pro Plus and quantified using Quantity One software (Bio-Rad) as previously described [27,28]. The hybridization signals of specific mRNAs were normalized to that of 18S RNA in each sample.

### Data presentation and analysis of results

Contractile responses were expressed as maximal wall tensions (mN/mm) and as a negative logarithm of the agonist concentration producing 50% of maximal wall tension (pD_2_). Relaxations were depicted as percentage of pre-existing contraction. Statistical comparisons were carried out using one-way and two-way analyses of variance (ANOVA), and the Tukey test was used for post-hoc analyses (SPSS 17.0, SPSS Inc., Chicago, IL, USA). ANOVA for repeated measurements was applied when data consisted of repeated observations at successive observation points. The results were expressed as mean ± SEM and P < 0.05 denoted statistical significance. Unless otherwise indicated, the *P* values refer to one-way ANOVA.

### Drugs

The drugs used in the present study were: ketamine (Parke-Davis Scandinavia AB, Solna, Sweden), cefuroxim, diazepam (Orion Pharma Ltd., Espoo, Finland), metronidazole (B. Braun AG, Melsungen, Germany), buprenorphine (Reckitt & Colman, Hull, England), acetylcholine chloride, norepinephrine bitartrate, L-NAME hydrochloride, NS-1619 (Sigma-Aldrich Chemical Co, St Louis, MO, USA), sodium nitroprusside (Fluka Chemie AG, Buchs SG, Switzerland). Stock solutions of the compounds used in the functional arterial experiments were made by dissolving the compounds in distilled water. Solutions were freshly prepared before use and protected from light.

## Results

### Blood pressure, body weight, and heart weight

Systolic BP did not differ in the study groups at the beginning of the oxonic acid diet (Table 1). At the end of the study, mean systolic BP was higher in the two NX groups versus Sham groups (P = 0.041, two-way ANOVA), and concomitantly increased right and left ventricular weights were also observed. Hyperuricemia did not influence BP or ventricular weights, but was associated with lower final body weight (P = 0.004, two-way ANOVA).

**Table 1:** **Experimental group data (oxonic acid feeding period from week 3 to 12)**

	**Sham**	**Sham + Oxo**	**NX**	**NX + Oxo**
Systolic blood pressure				
Week 3 (before treatment)	119 ± 4	120 ± 5	128 ± 5	127 ± 5
Week 12	133 ± 7	135 ± 5	141 ± 6^‡^	151 ± 5^‡^
Body weight (g)				
Week 3	341 ± 6	337 ± 8	335 ± 8	331 ± 7
Week 12	436 ± 9	411 ± 12^#^	452 ± 11	412 ± 30^#^
Heart weight (g/kg) at the end of study				
Right ventricle	0.28 ± 0.01	0.29 ± 0.01	0.34 ± 0.03^‡^	0.33 ± 0.03^‡^
Left ventricle	1.71 ± 0.06	1.86 ± 0.06	2.23 ± 0.11* ^‡^	2.41 ± 0.18* ^‡^
Removed kidney tissue (g/kg)			7.67 ± 0.17	7.50 ± 0.08
Uric acid (μmol/l)	33.5 ± 11.8	121.6 ± 22.4*	64.4 ± 21.0	156.7 ± 20.2* ^†^
Creatinine (μmol/l)	40.1 ± 5.7	49.3 ± 3.2	80.7 ± 3.2*^‡^	81.8 ± 8.8*^‡^
Urea (mmol/l)	6.5 ± 0.4	8.3 ± 0.5	12.9 ± 0.6*^‡^	14.3 ± 2.2*^‡^

Values are mean ± SEM; *n* = 11, except for laboratory values in the Sham group *n* = 9. *P < 0.05 compared with the Sham group, ^†^ *P* < 0.05 compared with the NX group using one-way ANOVA; ^‡^ *P* < 0.05 NX groups compared with the Sham groups using two-way ANOVA; ^#^ *P* < 0.05 Sham+Oxo and NX+Oxo groups compared with the Sham and NX groups using two-way ANOVA.

### Laboratory findings

Plasma UA was 2.4-fold elevated in the Sham+Oxo and 3.6-fold elevated in the NX+Oxo rats when compared with the corresponding controls (p = 0.005 and p = 0.002, respectively), while subtotal renal ablation did not significantly influence plasma UA levels (Table 1). Plasma creatinine and urea levels were elevated approximately two-fold in the NX groups and were not affected by hyperuricemia.

### Ventricular load, as evaluated using ANP, BNP, SkαA and β-MHC mRNA levels

**Figure 1:**
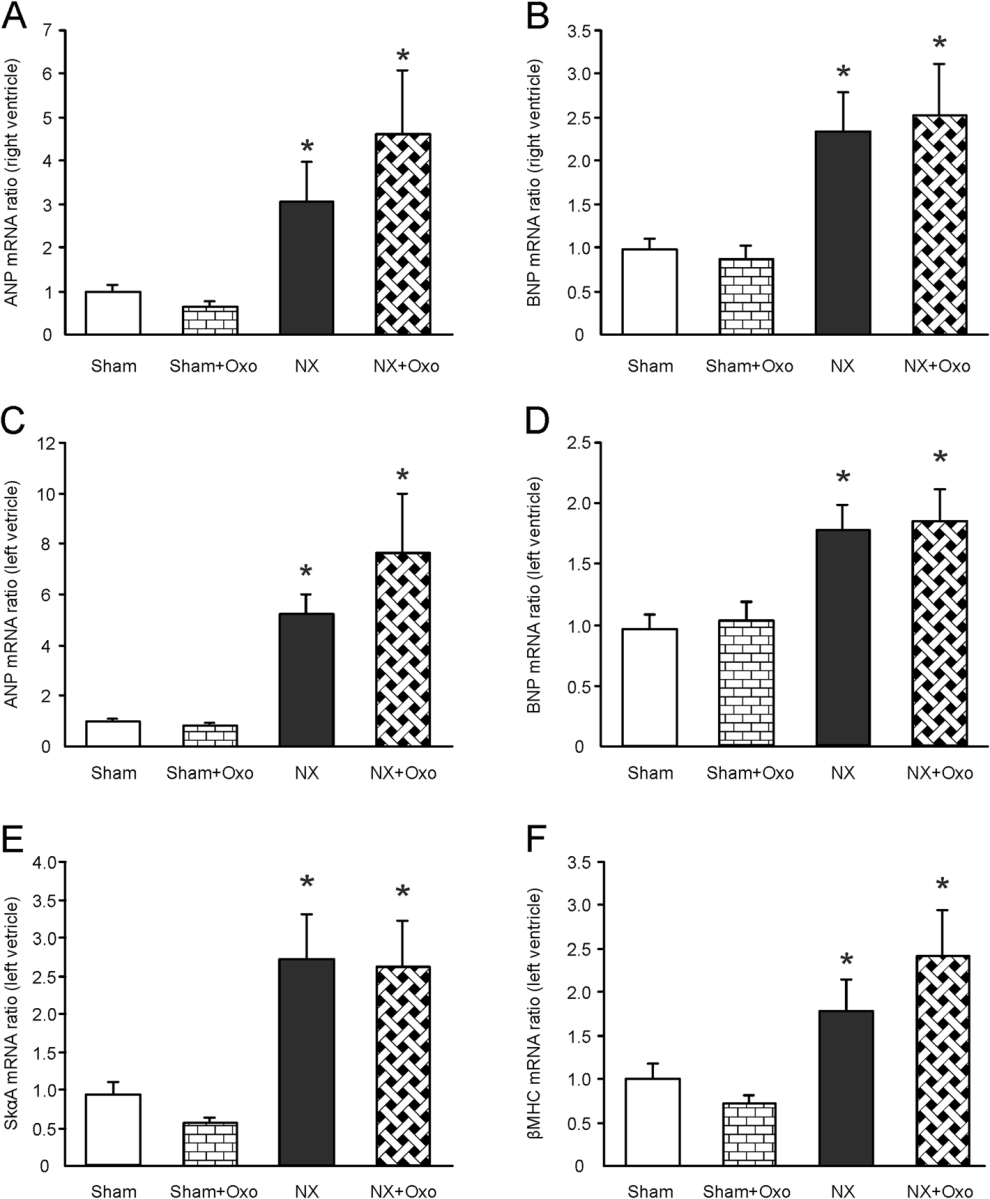
***Evaluation of cardiac load.*** Right ventricular atrial natriuretic peptide (ANP) **(A)** and B-type natriuretic peptide (BNP) **(B)**; and left ventricular ANP **(C)**, BNP **(D)**, skeletal α-actin (SkαA) **(E)**, and β-myosin heavy chain (β-MHC) **(F)** mRNAs in the experimental groups; values are mean±SEM, *n* = 11 for all groups; *P < 0.05, two-way ANOVA compared with the Sham groups. NX=5/6 nephrectomized rat, Sham=sham-operated rat, Oxo=2.0% oxonic acid diet.

The synthesis of right and left ventricular ANP and BNP mRNAs, and left ventricular SkαA and β-MHC mRNAs, were clearly higher in the NX groups when compared with the Sham groups (P < 0.05 for all, two-way ANOVA) (Figure 1). Oxonic acid feeding did not influence the mRNA levels of these genes.

### Functional responses in the main branch of the mesenteric artery

**Figure 2:**
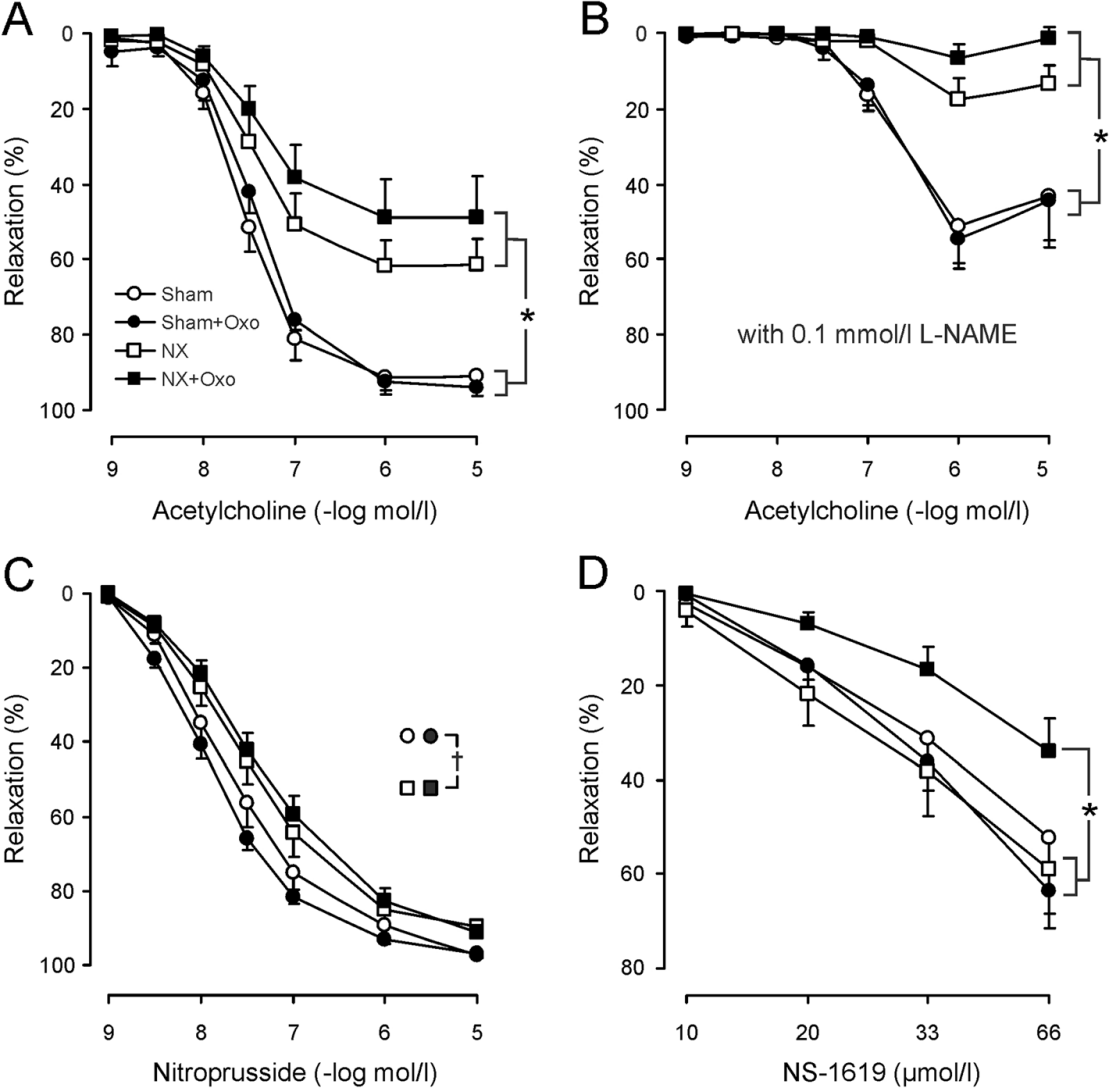
***Vasorelaxation in vitro.*** Line graphs show relaxation responses induced by acetylcholine in the absence **(A)** and presence **(B)** of L-NAME, and relaxations elicited by the NO donor nitroprusside **(C)**, and the large conductance calcium-activated potassium channel opener NS-1619 **(D)** in the experimental groups; values are mean ± SEM, *n* = 10 for all groups; *P < 0.05, ANOVA for repeated measurements; ^†^ *P* < 0.05 NX groups compared with the Sham groups using two-way ANOVA for repeated measurements. Groups as in Figure 1.

Contractile sensitivity to NE was slightly higher in the NX groups when compared with the two Sham groups (P < 0.001, two-way ANOVA). However, the difference in the contractions to NE did not curtail the results on vasorelaxation, as the relaxations were elicited after 5 μM NE concentration that induced approximately 70-80% of maximal response in all groups. Responses to KCl were comparable between NX and Sham rats. The relaxation induced by Ach in the main branch of the mesenteric artery was markedly impaired in the NX groups, while experimental hyperuricemia did not influence these responses either in Sham or NX rats (P = 0.208 NX versus NX+Oxo groups, ANOVA for repeated measurements) (Figure 2A). Inhibition of NOS with L-NAME equally reduced the relaxation to Ach in the Sham and Sham+Oxo groups, while the response was almost abolished in the NX and NX+Oxo groups (Figure 2B). As displayed by the diminished relaxation to NP, vasorelaxation via cGMP in arterial smooth muscle was reduced in the NX groups when compared with the Sham groups (P = 0.008, two-way ANOVA for repeated measurements), whereas oxonic acid feeding did not influence this response (Figure 2C). In contrast, vasorelaxation induced by the BK_Ca_ opener NS-1619 was significantly reduced in NX+Oxo rats when compared with all other groups (P = 0.032, ANOVA for repeated measurements) (Figure 2D).

### Functional responses and morphology of the small mesenteric artery

**Figure 3:**
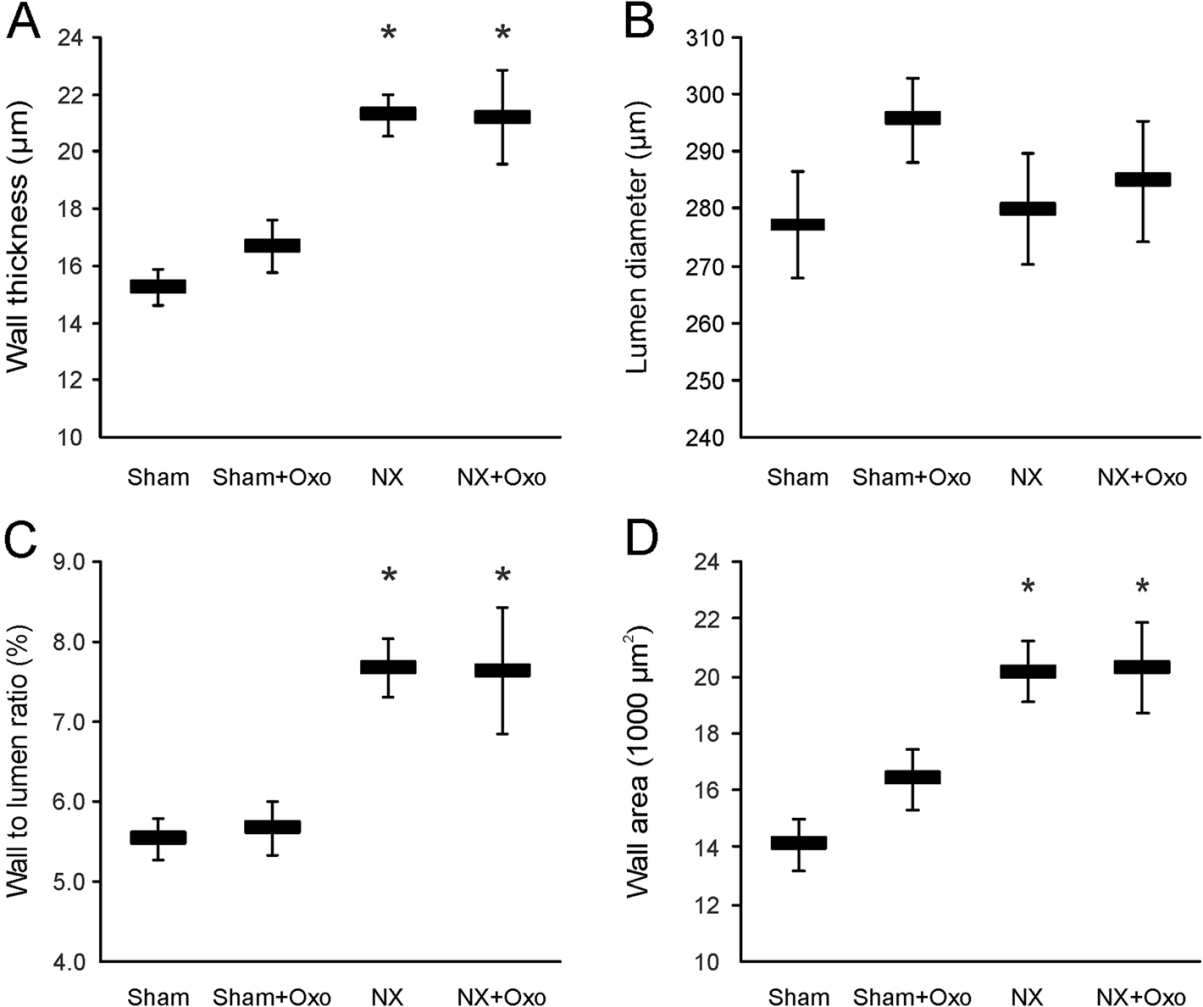
***Arterial morphology.*** Small artery wall thickness (μm) **(A)**, lumen diameter (μm) **(B)**, wall to lumen ratio (%) **(C)**, and wall area (μm^2^) **(D)** at 90 mmHg; values are mean ± SEM, *n* = 10 for all groups; *P < 0.05 versus Sham. Groups as in Figure 1.

In the small arterial rings, maximal wall tension in response to NE was higher in the NX rats than in Sham rats (P = 0.03, two-way ANOVA), while the sensitivity to NE (pD_2_) was similar in all groups (Table 2). Responses to KCl were comparable between NX and Sham rats also in the small mesenteric artery. Isolated second order mesenteric artery branches from NX rats exhibited hypertrophic remodeling: increased wall thickness, wall to lumen ratio, and wall cross-sectional area without changes in lumen diameter when compared with Sham rats. Experimental hyperuricemia did not significantly influence the structure of small mesenteric artery (Figure 3).

**Table 2:** **Parameters of contractile responses of isolated second order branches and the main branch of the mesenteric artery**

	**Sham**	**Sham+Oxo**	**NX**	**NX+Oxo**
**Small artery**				
*Norepinephrine*				
pD_2_ (−log mol/l)	5.81 ± 0.08	6.00 ± 0.09	5.81 ± 0.09	5.87 ± 0.11
Maximal wall tension (mN/mm)	5.46 ± 0.17	5.82 ± 0.50	6.63 ± 0.54^‡^	6.58 ± 0.39^‡^
*KCl*				
pD_2_ (−log mol/l)	1.41 ± 0.01	1.42 ± 0.02	1.39 ± 0.02	1.39 ± 0.03
Maximal wall tension (mN/mm)	5.59 ± 0.43	5.93 ± 0.56	6.37 ± 0.49	6.84 ± 0.32
**Main branch**				
*Norepinephrine*				
pD_2_ (−log mol/l)	6.26 ± 0.14	5.97 ± 0.05	6.49 ± 0.13^‡^	6.64 ± 0.11^‡^
Maximal wall tension (mN/mm)	7.30 ± 0.49	8.60 ± 0.49	9.14 ± 0.63	9.64 ± 1.52
*KCl*				
pD_2_ (−log mol/l)	1.51 ± 0.02	1.51 ± 0.03	1.51 ± 0.03	1.53 ± 0.03
Maximal wall tension (mN/mm)	6.79 ± 0.85	7.01 ± 0.94	7.53 ± 0.67	8.02 ± 0.96

Values are mean ± SEM, *n* = 10 for all groups. pD_2_ is the negative logarithm of the concentration of agonist producing 50% of the maximal response. ^‡^ *P* < 0.05 NX groups compared with Sham groups using two-way ANOVA.

## Discussion

Oxonic acid-induced hyperuricemia has been widely studied in recent years, but consensus on the putative harmfulness of increased circulating UA concentration still remains elusive. In this study we employed the 5/6 nephrectomy rat model of CRI to investigate the influence of experimental hyperuricemia on the tone and morphology of the mesenteric artery. The NX rats showed several characteristic findings of moderate renal insufficiency [16,29], whereas experimental hyperuricemia did not influence vasoconstrictor responses, renal function, cardiac load, or small artery morphology. To our knowledge, the present study is the first to suggest that hyperuricemia may impair vasorelaxation via BK_Ca_, indicating alteration in smooth muscle hyperpolarization.

Oxonic acid feeding inhibits the oxidation of UA to its metabolite, allantoin, resulting in hyperuricemia. In the present study, oxonic acid diet elevated plasma UA 2.4 to 3.6-fold, which is in line with the 1.3 to 2.8-fold elevations observed in previous experimental studies [4-6]. The development of stage 2–3 renal insufficiency was confirmed by the elevated levels of plasma creatinine and urea, impaired endothelium-mediated vasodilatation, hypertrophic remodeling of the resistance vessels, and modest elevation of BP [15,29]. Increased right and left ventricular weights, higher ANP and BNP mRNA content, as well as increased left ventricular SkαA and β-MHC mRNA content, indicated permanent volume and pressure overload after subtotal renal ablation [15]. However, hyperuricemia did not significantly influence any of the indicators of cardiac load either in the Sham or NX rats.

Arterial contractions were examined here in order to reveal differences in vasoconstrictor sensitivity that would potentially interfere with the interpretation of the relaxation experiments. No differences were found in responses elicited by membrane depolarisation with KCl (Table 2). In the main branch, NX rats exhibited slightly higher sensitivity but no significant change in maximal response to NE. However, in the small artery the NX groups exhibited slightly higher maximal response without changes in sensitivity to NE. Importantly, oxonic acid feeding did not influence the vasoconstrictor responses either in the second order or main branches of the mesenteric artery. Thus, changes in the vasodilator responses induced by hyperuricemia were not explained by alterations in vasoconstrictor responses.

Oxonic acid feeding did not induce changes in the morphology of small mesenteric arteries, while hypertrophic arterial remodeling was clearly observed in the NX rats [15]. Previously, oxonic acid diet was found to activate renal RAS, as indicated by increased expression of juxtaglomerular renin, and result in hypertrophic remodeling of the glomerular afferent arterioles [5-7,30]. Although local vascular RAS components were not examined here, the oxonic acid model of hyperuricemia is characterized by elevated plasma levels of aldosterone with subsequent sodium retention [8]. The present results suggest that despite possible activation of the circulating RAS, high UA level does not influence the morphology of small arteries and heart.

Vasorelaxation was investigated in the main branch of the mesenteric artery, which functionally resembles the second-order branches in the same arterial bed [15,16]. Unlike the rat aorta, where endothelium-dependent vasodilatation is largely mediated via NO, the endothelium-derived relaxation of the mesenteric artery is also mediated by hyperpolarization of smooth muscle [15]. Relaxation to Ach was impaired in NX rats, and the response was practically abolished in the presence of L-NAME in both NX groups, indicating that it was mediated via NO. Inhibition of NOS reduced the relaxation to Ach also in Sham rats, but the vessels showed more pronounced relaxations in the presence of L-NAME than those from NX rats [15,16]. The L-NAME resistant vasorelaxation in the rat mesenteric artery has been attributed to endothelium-dependent hyperpolarization [15-17]. In Sham and NX rats hyperuricemia was without significant influence on the response to Ach, indicating that endothelium-dependent relaxation was not affected by oxonic acid feeding. Vasorelaxation to the exogenous NO donor NP was slightly impaired in the NX groups when compared with the Sham groups, but hyperuricemia did not influence the sensitivity of arterial smooth muscle to relaxation via cGMP.

The present study showed that the endothelium-independent vasodilatation induced by the BK_Ca_ channel opener NS-1619 was impaired in the NX+Oxo group. Notably, this impairment was not observed in Sham+Oxo rats. NS-1619 induces relaxation by triggering intracellular Ca^2+^ sparks, which induce K^+^-efflux via BK_Ca_ and lead to subsequent hyperpolarization [31-33]. The finding of decreased vasorelaxation sensitivity via BK_Ca_ solely in the hyperuricemic NX rats suggests that the effect was specifically associated with the combination of uremic milieu and increased plasma UA concentration.

Several mechanisms could result in alterations of BK_Ca_ mediated vascular tone. The BK_Ca_ channel, which consists of α- and β1-subunits, is the most prominent type of calcium-activated K^+^ channel in arterial smooth muscle [13]. The β1-subunit is responsible for tuning the Ca^2+^-sensitivity [34]. Interaction between the α-subunit and β1-subunit enhances Ca^2+^-sensitivity of BK_Ca_ channels, whereas the loss of the β1-subunit decreases Ca^2+^ sensitivity [35]. In a recent study using diabetic mice [36], BK_Ca_ expression in arterial myocytes was strongly influenced by the calcineurin pathway, which inhibits the expression of the regulatory β1-subunit.

Endogenous BK_Ca_ inhibitors may influence K^+^-channel activity. Arachidonic acid metabolites, especially 20-hydroxyeicosatetraenoic acid (20-HETE), can inhibit BK_Ca_ [37]. 20-HETE reduces the open-state probability of the channel [38]. Another endogenous inhibitor of BK_Ca_ is hydrogen sulfide, which binds to the α-subunit and increases the voltage needed for channel activation [39]. BK_Ca_ are also modulated by reactive oxidative species (ROS), which can activate or inactivate BK_Ca_ [13]. Such mechanisms are relevant, as elevated UA level following 2.0% oxonic acid feeding has increased total peroxyl radical-trapping capacity and reduced oxidative stress markers in the rat [14]. Hyperuricemia may increase superoxide dismutase (SOD) activity [40], which catalyzes the dismutation of superoxide (O_2_^−^) into oxygen and hydrogen peroxide (H_2_O_2_). UA itself is able to scavenge BK_Ca_-inhibiting radicals, and increase the production of H_2_O_2_ by preventing the H_2_O_2_-induced inactivation of SOD [40]. H_2_O_2_ can even induce vasodilatation directly via BK_Ca_ activation [41], an effect known to be more pronounced under conditions of reduced NO availability [13]. The latter is a characteristic feature of the uremic milieu [42]. Taken together, a multitude of processes can influence vasorelaxation via BK_Ca_, including changes in channel protein gene expression and structure, changes in cellular Ca^2+^ sparks, levels of ROS, and endogenous BK_Ca_ inhibitors.

UA is produced from xanthine by the enzyme xantine oxidase, which has been found to play an important role in a variety of tissue and vascular injuries [43]. Although therapeutic interventions with the aim to lower UA with xanthine oxidase inhibitors may be beneficial in treating the vascular disorders associated with renal disease, debate is still ongoing whether the effect is related to lowering UA levels *per se*, or to reduced xanthine oxidase activity. The present protocol did not include the treatment of hyperuricemia, since the UA-lowering drugs allopurinol, febuxostat and uricosuric agents have been well documented to prevent the pathophysiological changes induced by the oxonic acid feeding [4-6,10,30,44-46].

## Conclusions

We show here that 2.0% oxonic acid diet increased plasma UA, but did not significantly influence BP, resistance vessel structure, and cardiac load as evidenced by the unaltered ventricular weights and mRNA levels of natriuretic peptides, SkαA, and β-MHC. Hyperuricemia did not influence endothelium-dependent NO-mediated vasorelaxation, but oxonic acid feeding impaired vasorelaxation elicited by the BK_Ca_ channel opener NS-1619 in this model of CRI. Future studies are needed to define the molecular mechanisms by which hyperuricemia can influence BK_Ca_ function in experimental CRI.

## Abbreviations

20-HETE: 20-hydroxyeicosatetraenoic acid
Ach: Acetylcholine
ANOVA: Analysis of variance
ANP: Atrial natriuretic peptide
BK_Ca_: Ca^2+^-activated K^+^-channel
β-MHC: ß-myosin heavy chain
BNP: B-type natriuretic peptide
BP: Blood pressure
CRI: Chronic renal insufficiency
L-NAME: N^G^-nitro-L-arginine methyl ester
NE: Norepinephrine
NO: Nitric oxide
NOS: Nitric oxide synthase
NP: Nitroprusside
NX: 5/6 nephrectomy
Oxo: 2.0% oxonic acid diet
RAS: Renin-angiotensin system
ROS: Reactive oxygen species
SkαA: Skeletal α-actin
UA: Uric acid
